# In-Office Biceps Tenotomy with Needle Arthroscopy: A Feasibility Study

**DOI:** 10.1016/j.eats.2021.01.022

**Published:** 2021-04-12

**Authors:** Marc-Olivier Gauci, Brieuc Monin, Alexandre Rudel, Laurent Blasco, Bastien Bige, Pascal Boileau

**Affiliations:** Institut Universitaire Locomoteur & Sport, Unité de Recherche Clinique Côte d’Azur (UR2CA), Hôpital Pasteur 2, Université Côte d’Azur, Nice, France

## Abstract

Isolated pathology of the long head of the biceps is an indication for biceps tenotomy. To date, needle arthroscopy allows a direct diagnosis of shoulder lesion. We aimed to evaluate the technical feasibility of an in-office biceps isolated tenotomy by needle arthroscopy. Advantages were found in the fast-track process and the high rate of satisfaction in our selected patients. It was also a way to correct the diagnosis of torn biceps missed by the imaging. However, performing this procedure requires previous experience in conventional arthroscopy and should not be performed on anxious patients. Further studies will be necessary to confirm the reproducibility of this promising method, which could be a valuable alternative to heavy in-operating room process.

Tendinopathy and instability of the long head of the biceps (LHB) are well-known isolated etiologies of chronic shoulder pain.^1^ The most common arthroscopic treatment consists of removing the intra-articular portion of the LHB and performing tenotomy with or without tenodesis in the bicipital groove. The literature reports little advantage in tenodesis giving less biceps deformity (Popeye sign), especially in young patients; however, this technique requires a longer operating time, greater implant costs, and sometimes more complications. Thus, it appears that isolated biceps tenotomy could be an interesting option with the same benefit on pain,^2^ especially in older or nonathlete patients with earlier improvement in postoperative pain.^3^ Multiple attempts have been made to propose an in-office surgery to avoid the heavy process of in-operating room (OR) procedure and bring faster satisfaction to the indicated patients. Clinical ultrasound-guided biceps tenotomy series have been published with cases of cuff lacerations, cartilage injury, and up to 75% of incomplete tenotomy, even with an arthroscopic hook blade through a deep to superficial approach. Moreover, persistence of a proximal and hypertrophic stump (“hourglass biceps”^4^) into the glenohumeral joint could induce mechanical locking and discomfort. Recently, in-office needle arthroscopic diagnoses were made possible with the miniaturization of optic devices for knee and shoulder,^5^, ^6^, ^7^ and therapeutic procedures such as partial meniscectomy also were made possible. The purpose of the present study is to assess the feasibility of in-office biceps tenotomy (IOBT) with a needle arthroscopy.

## Surgical Technique (With Video illustration)

### Indications

We assume the IOBT procedure would be efficient in the following specific indications: massive, irreparable rotator cuff tears with remaining shoulder pain and biceps still present; isolated biceps pathology with intact rotator cuff, particularly in patients with tenosynovitis, subluxation, prerupture, or a SLAP lesion; and failed cuff repair with the biceps pulley in place but pathologic. However, some contraindications must be evocated: an active patient with heavy work activity, athletes and muscled patients, frozen shoulder, anticoagulant or antiplatelet medication, infection, recent fracture, and refusal (anxiety, esthetic issue). An ethical agreement was obtained (institutional research board: 2020-SH01-01)

### Patient Position and Office Organization

Patients were positioned in a beach-chair position to keep their legs raised. The temperature of the office was kept around 20°C. Patients were not scoped. We preferred the supine position instead of the lateral decubitus to facilitate the decoaptation of the humeral head and avoid the closing of the glenohumeral joint space by the falling of the humeral head on the glenoid. The shoulder was fully offset outside the table with no other support. An assistant helped at any time carrying out a slight traction or small rotations.

**Fig 1:**
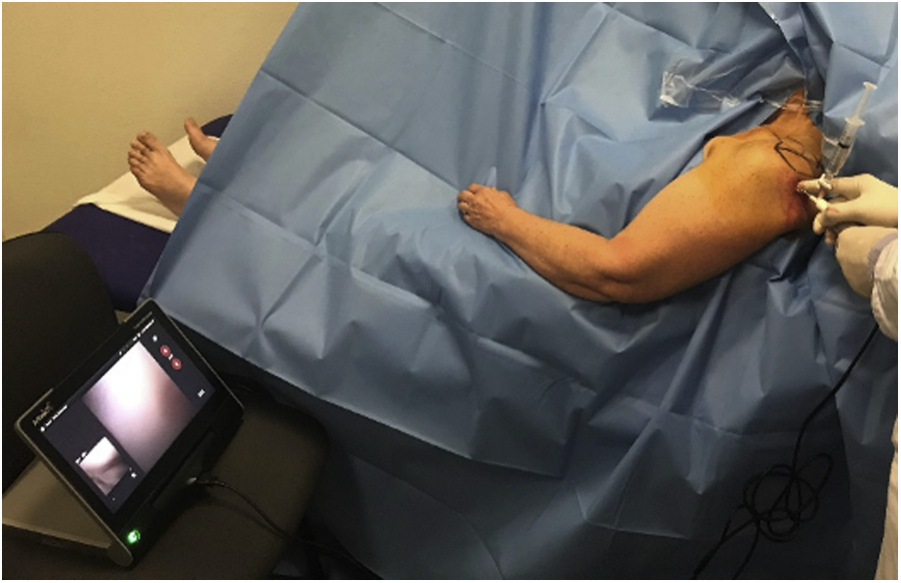
Contrary to in-operating room procedure, the position of the screen during in-office biceps tenotomy has to be low to have a direct and comfortable view of it.

The screen was placed in front of the surgeon, under the level of his waist (Fig 1). Patient shoulder asepsis was performed with 2 consecutive Betadine chains. The shoulder was then prepared with a surgical drape to separate the patient from the procedure site. Anatomical landmarks and 2 anterior and posterior approaches were finally drawn similarly to the classic arthroscopic approaches.

**Fig 2:**
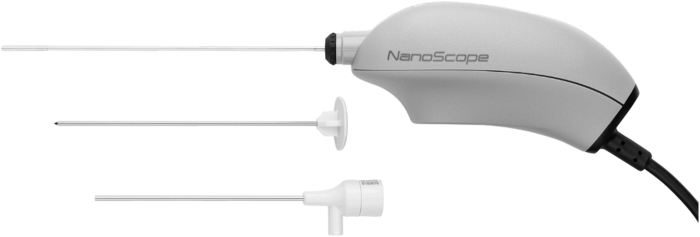
The needle arthroscopic device (NanoScope; Arthrex) is a needle arthroscopy device with a 1.9-mm scope. Technical characteristics: 400 × 400 resolution, 120-degree field of view, auto-focus from 3 mm × 100 mm. The device has a zero-degree viewing angle.

The needed material was the needle arthroscopic device jig (NanoScope; Arthrex, Naples, FL, Fig 2), a conventional arthroscopic scissor, an 11-blade scalpel, 2-L locked-syringe, a sterile bowl, 2 L of physiological serum, a lumbar puncture needle, 80 mL of Lidocaine 1% with added adrenaline, a whitewash kit, and a dermographic pencil.

### Procedure

**Fig 3:**
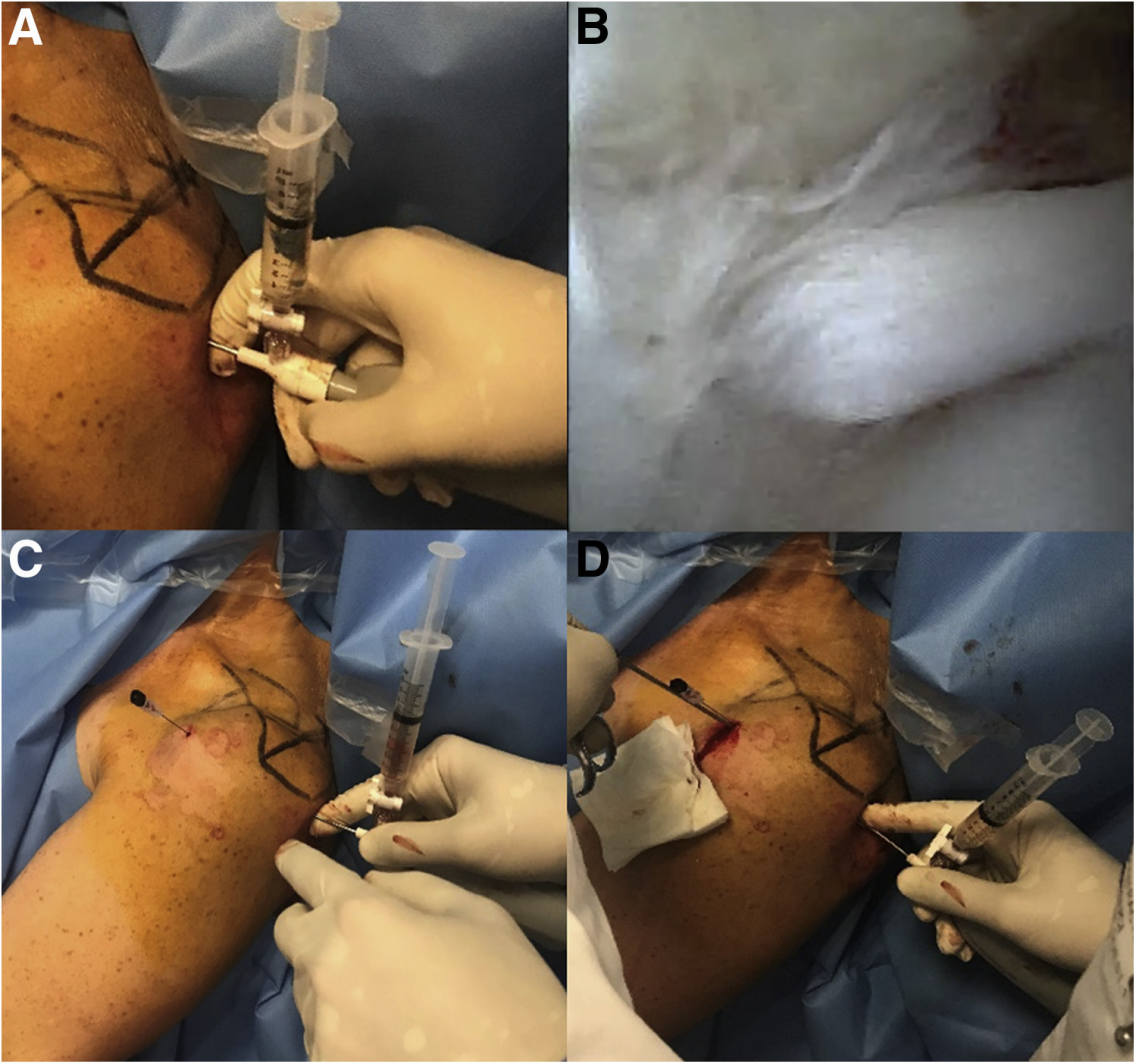
Diagnostic step and approaches for in-office biceps tenotomy. (A) The needle arthroscopy is introduced from a posterior approach. (B) The pathologic biceps and the pulley lesion are visualized and confirmed the diagnosis. (C) A needle is interiorly positioned to guide the anterior approach through the rotator cuff interval. (D) The scissors are introduced through the rotator cuff interval.

The needle arthroscopy procedure begins with a posterior approach (Video 1). Local anesthesia is performed with Lidocaine 1% from the skin to the capsule. The introducer and the scope are slightly introduced in the glenohumeral joint (Table 1). The patient needs to be reassured to avoid any shoulder contraction that would narrow the glenohumeral joint. Local anesthesia completion is possible if necessary. The most sensitive part in the whole procedure is the joint capsule. The exploration step allows a biceps and a cuff evaluation (Fig 3).

**Table 1:** Pearls and Pitfalls

Criteria	Pearls	Pitfalls
Installation	Patient must be comfortable with the shoulder outside the stretcher, a pillow could be position under the head, legs up. Temperature in the room must be cool.	Procedure can take few minutes and patient must be relaxed to facilitate the procedure. Vasovagal syncope should be prevented.
Posterior approach	A routine posterior soft point approach is used.	The posterior approach must lead at the upper part of the glenohumeral joint to reach the biceps.
Scope introduction	Shoulder must be in internal rotation, patient relaxed and warned at each step to keep him confident.	Entering the shoulder by the posterior soft point is more difficult than under general anesthesia. Internal rotation with slight traction allows to open the posterior space and to stretch the posterior capsula then to go through it.
Anterior approach	A needle must be used to determine the anterior approach.	Anterior portal should allow a perpendicular access to the biceps to be the most efficient in cutting it.
Scissors	Conventional arthroscopic scissors (instead of nanoinstruments) must be used to cut the biceps.	The biceps is often hypertrophic then adapted scissors are needed.
Biceps tenotomy	Tenotomy must be performed by pushing the biceps at the same time with the elbow in extension.	Biceps tendon can slide when cutting it. Extension of the elbow helps stabilize it.

**Fig 4:**
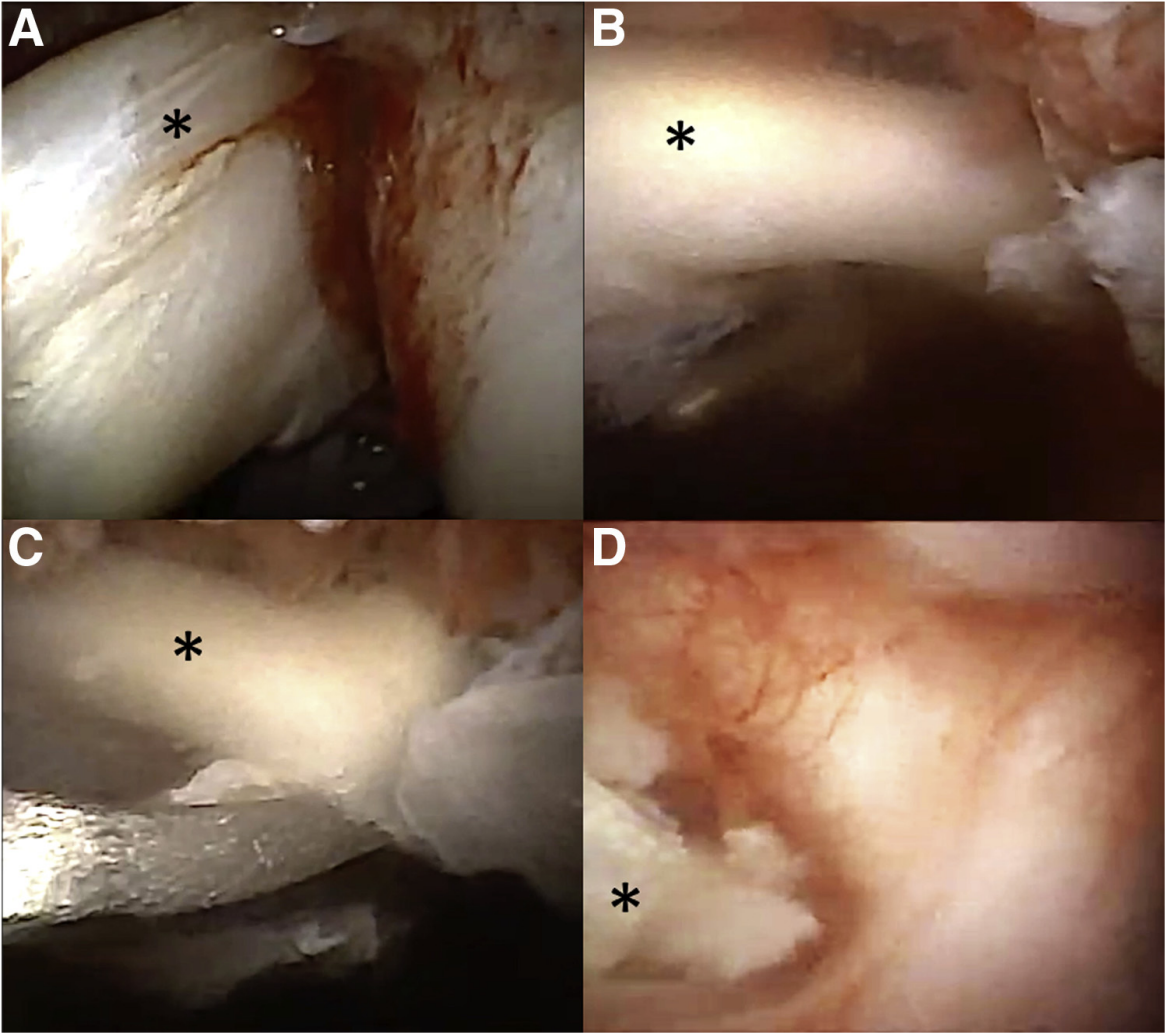
Tenotomy of the biceps. (A) Pathologic biceps observation under air needle-arthroscopy. (B) Same pathologic biceps observed after intra-articular serum injection. (C) Conventional arthroscopic scissors are introduced through the anterior approach to perform the biceps tenotomy. (F) Final result with the biceps locked in the bicipital groove. ∗ indicates biceps.

One of the key points of the technique is to inject air into the joint to allow a high-quality visualization of the joint structures to perform static and dynamic testing of the biceps. When needed, we added physiological serum with lidocaine and adrenaline to limit the bleeding and pain. After confirmation of a pathologic biceps and tenotomy indication, the anterior approach was localized with a lumbar puncture needle through the rotator interval. A second anesthesia was carried out for the anterior portal, and a skin incision allowed to introduce the scissors in the shoulder. We used conventional arthroscopic scissors as the biceps was often hypertrophic (Fig 4). Exchange of fluids (regular aspiration-injection) through the locked syringe allowed us to limit the bleeding and improve intra-articular visualization.

**Fig 5:**
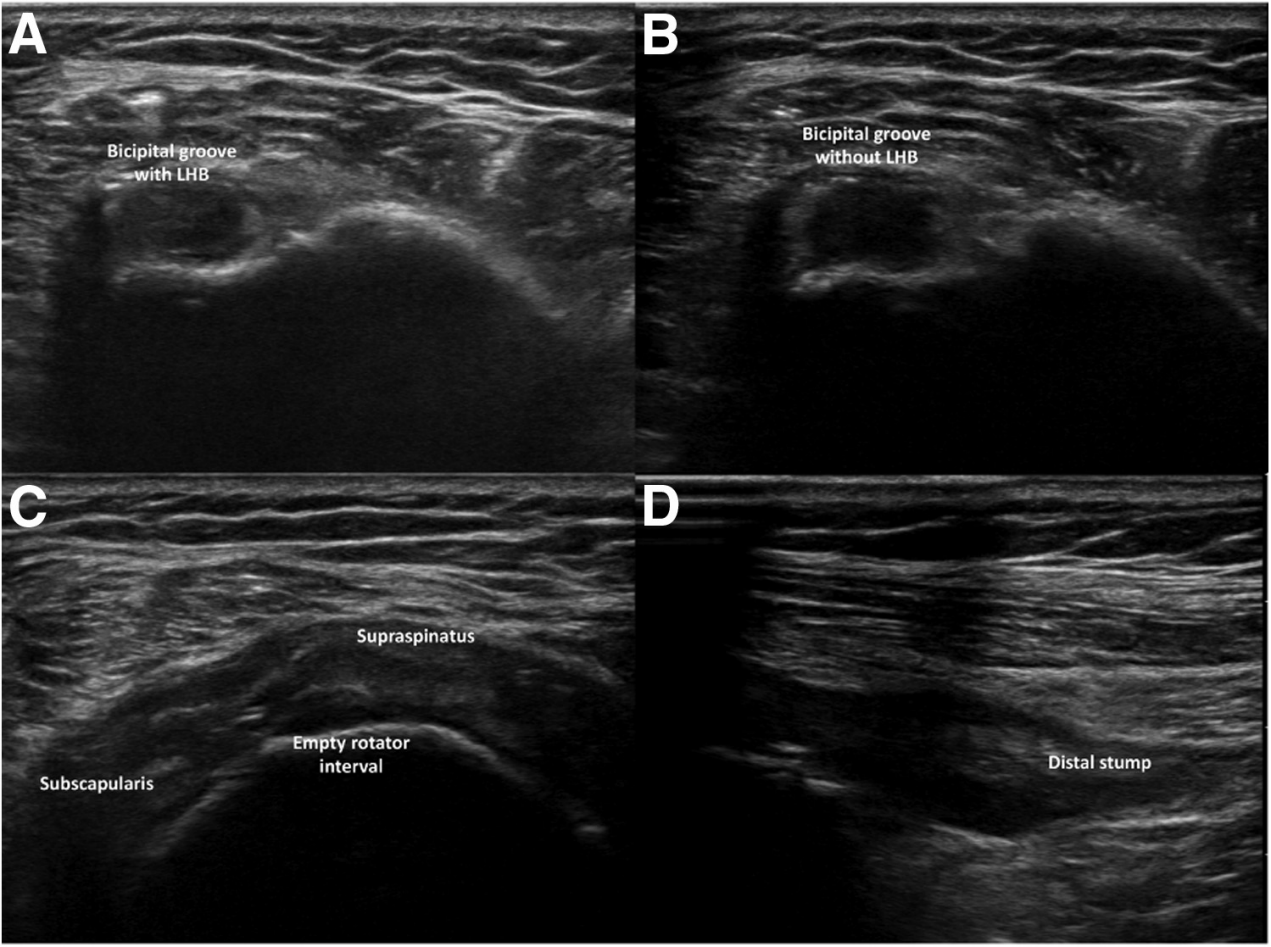
Ultrasound check before (A) and after in-office biceps tenotomy: the groove is empty (B, C) and the biceps stump is found more distally (D). (LHB, long head of the biceps.)

**Fig 6:**
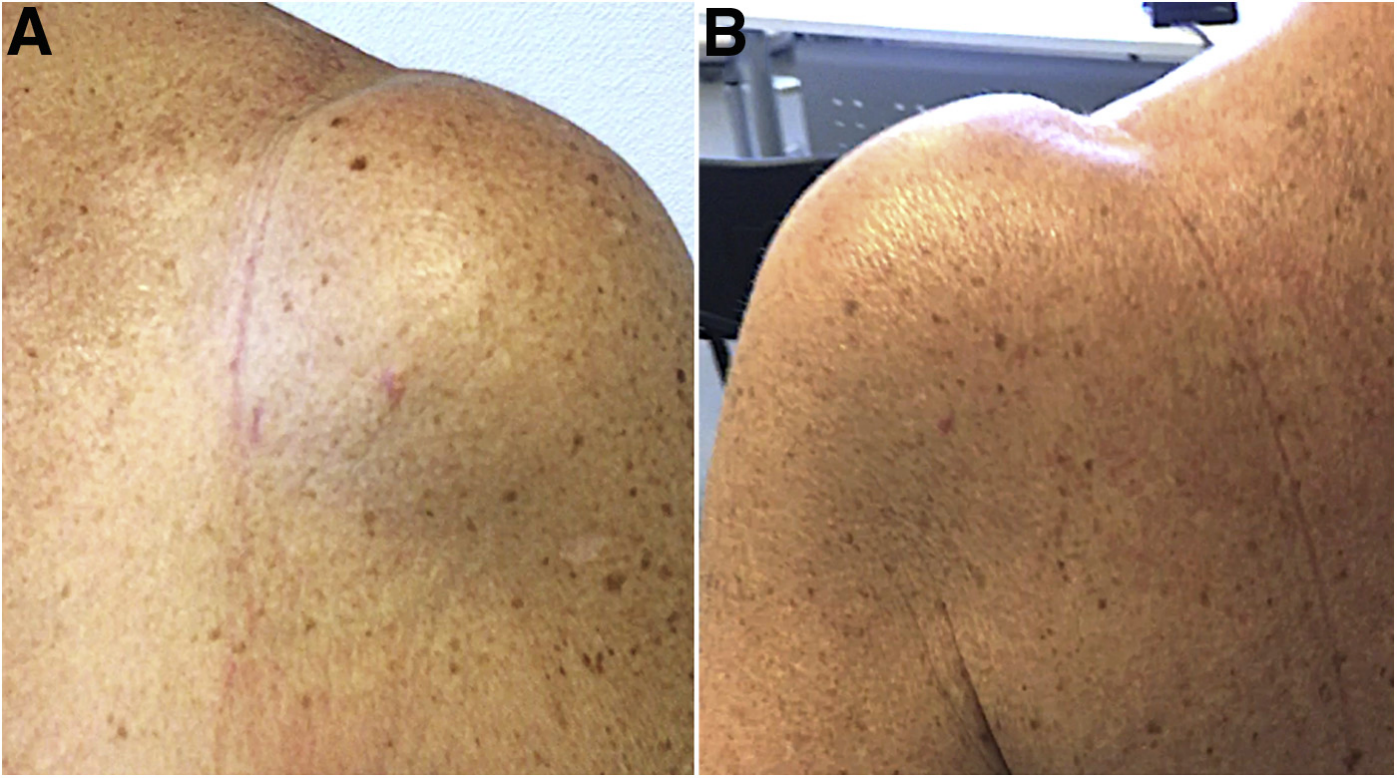
Skin aspect at 1 week after in-office biceps tenotomy. Scares are small or not visible (anterior [A], posterior [B]).

After IOBT, a subacromial infiltration was made with a one-shot corticoid injection, simple bandage covers the 2 incisions, and a slightly tight bandage surrounded the arm to prevent from the Popeye sign. We also carried out an ultrasound control of the distal stump in the groove after the tenotomy (Fig 5). Scars were no more visible in the 2 to 3 weeks after the procedure (Fig 6).

## Discussion

The main finding of this study is that IOBT with needle arthroscopy procedure is technically feasible. However, there is a learning curve with some possible failures due to difficulties in having a clear intra-articular vision and intraoperative pain control in anxious patients. We carried out this technique on 6 patients with 4 successful results, notably fast and satisfying postprocedure results on pain. A conversion to a classical biceps tenotomy under arthroscopy was needed in 1 patient due to uncontrolled pain in an anxious patient. The last patient had an already-torn biceps not detected on magnetic resonance imaging or ultrasound. Another interesting finding is that intra-articular injection of air provides a better vision than with physiological serum. Ideally, the IOBT could be performed with air only. Surgeons should also be aware that the intra-articular vision given by the 0° nanoscope is slightly different than the one obtained with a classical 30° arthroscope. A bit of time is needed to adapt. This is especially the case for approaches that could be slightly modified, and we recommend using a lumbar puncture needle for a better positioning.

We found many possible advantages on this needle arthroscopic procedure, but rigorous steps must be respected (Table 2). Under those conditions, we proposed a reliable and reproductible technique of tenotomy under needle arthroscopy. Contrary to ultrasound, IOBT with needle arthroscopy allowed for an intra-articular tenotomy of the LHB and direct visual control of the complete tenotomy. Then, there were no partial section, no persistence of a bothering intra-articular portion of a hypertrophic biceps, and no lesion of the subscapularis, as each step was thoroughly controlled.^8^

**Table 2:** Advantages and Disadvantages

Criteria	Advantages	Disadvantages
Diagnosis/procedure	Confirmation/correction of the imaging diagnosis of pathologic or torn biceps under awake procedure	Indication of isolated tenotomy should be accurate as exposed above respecting well-defined and previously published indications^8^
	Direct visualization of the intra-articular joint structures (cuff, labrum, cartilage). Correction of misdiagnosis on imaging.	
	Direct control of the completed tenotomy	
Patient experience	Fast process: no anesthesia consultation, no occupation of the operating theater, no outpatient surgery department needed	Anxious patients must not be included and could be a cause of failure
	Quick recovery after in-office procedure	
Surgeon/institution	Time saving Cost decrease	A previous great experience of conventional in-OR arthroscopy procedure should be recommended for the surgeon
Safety	Safe anesthetic procedure	Patients have to be well informed
	Safe surgical procedure	Asepsis procedure must be respected to limit any theoretical risk of infection

OR, operating room.

Further studies must be performed to specify the cost savings of the IOBT procedure and compensate the cost of a single-use camera. Moreover, larger studies must determine the integration of this technic in a daily activity and its acceptance by the patient.

In conclusion, IOBT with needle arthroscopy is technically feasible, although quite difficult with a learning curve, and we recommend a previous experience in conventional in-OR procedure. It is a safe procedure that can be performed on selected patients and offers a valuable alternative to a heavy OR process with potential advantages for the patient. Further studies with more patients involved will be necessary to confirm the reproducibility of this method.

## Supplementary Data

Video 1 This video summarizes our technique for in-office biceps tenotomy (IOBT) with needle arthroscopy. Patient matches the indications for biceps tenotomy. He is positioned in a comfortable beach chair position. A posterior approach allows to introduce the scope into the joint and to visualize the intra-articular pathologic biceps. No other lesions are observed. Conventional scissors are introduced through the rotator cuff interval and are used to cut the biceps at its insertion. At the end of the procedure, the biceps is visualized in its groove. The IOBT is carried-out under local anesthesia on a compliant and well-informed patient. Simple bandages finally cover the approaches.

ICMJE author disclosure forms
